# Spontaneous biases enhance generalization in the neonate brain

**DOI:** 10.1016/j.isci.2024.110195

**Published:** 2024-06-07

**Authors:** Shuge Wang, Vera Vasas, Laura Freeland, Daniel Osorio, Elisabetta Versace

**Affiliations:** 1 School of Biological and Behavioural Sciences, Queen Mary University of London, London, UK; 2 School of Life Sciences, University of Sussex, Brighton, UK

**Keywords:** neuroscience, developmental biology, computing methodology

## Abstract

Inductive generalization is adaptive in novel contexts for both biological and artificial intelligence. Spontaneous generalization in inexperienced animals raises questions on whether predispositions (evolutionarily acquired biases, or priors) enable generalization from sparse data, without reinforcement. We exposed neonate chicks to an artificial social partner of a specific color, and then looked at generalization on the red-yellow or blue-green ranges. Generalization was inconsistent with an unbiased model. Biases included asymmetrical generalization gradients, some preferences for unfamiliar stimuli, different speed of learning, faster learning for colors infrequent in the natural spectrum. Generalization was consistent with a Bayesian model that incorporates predispositions as initial preferences and treats the learning process as an update of predispositions. Newborn chicks are evolutionarily prepared for generalization, via biases independent from experience, reinforcement, or supervision. To solve the problem of induction, biological and artificial intelligence can use biases tuned to infrequent stimuli, such as the red and blue colors.

## Highlights

• Newborn chicks are evolutionarily prepared for generalization, via spontaneous biases
• Spontaneous generalization: biased, faster for some items, can include unfamiliar items
• Colors rare in the natural spectrum (red and blue) enhanced fast generalization
• In spontaneous generalization, initial preferences are updated at different rates

## Graphical abstract

**Figure:**
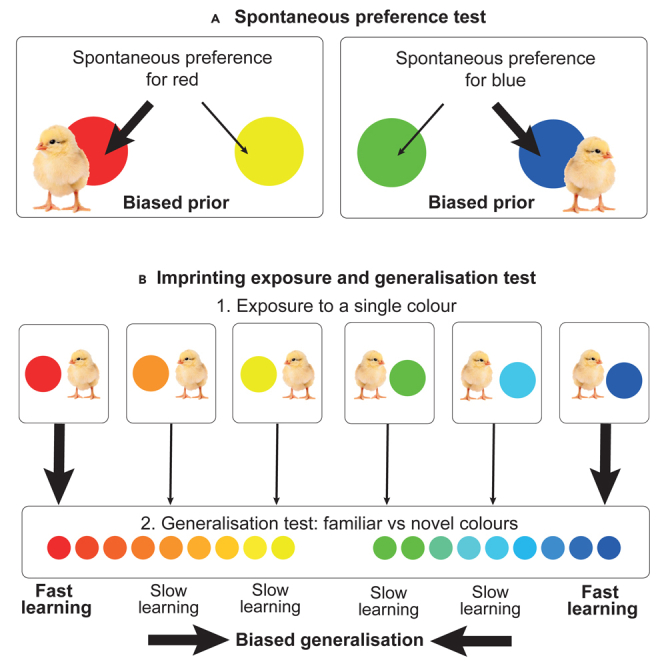

## Introduction

To respond adaptively to new challenges, animals must go beyond what they have experienced,^1^ from recognizing an individual from a novel point of view to predicting the actions of another individual. In particular inductive generalization, namely how animals extrapolate from limited previous evidence to new scenarios, is central to clarify the foundations of intelligent behavior and for the development of artificial intelligence.^2^ Therefore, a central question here is whether generalization has to build on expectations derived from direct experience or is supported by spontaneous biases (predispositions).

To investigate spontaneous generalization at the onset of life, we investigated poultry chicks, whose visual experience can be fully controlled from hatching until the moment of test.^3^^,^^4^ It has been observed that chicks are born with biases (called predispositions) that affect the learning rate of visual features, such as colors and shapes. For example, chicks learn to recognize a blue cube more quickly than a green hourglass^5^^,^^6^^,^^7^^,^^8^ (see in the study by Ono et al.^9^ for a parallel in quails), and colorful signals facilitate chicks in learning what prey is unpalatable.^10^ As previous work focused on rates of learning^11^^,^^12^^,^^13^^,^^14^ the question of whether spontaneous biases are essential to inductive generalization, in the absence of rewards and previous experience, remains unanswered. We therefore compared observed generalization with the predictions of an unbiased model which was derived from Shepard’s universal law of generalization.^15^ This law predicts that the probability of a response to one stimulus being generalized to another depends on the perceptual distance between them.^16^ This theory has been successfully applied^17^ to different stimuli (e.g., colors, tones, shapes) and taxa including mammals, birds, fish, amphibians, and insects.^18^

Filial imprinting^19^^,^^20^^,^^21^^,^^22^ is a rapid learning mechanism which allows newly hatched chicks to recognize and approach social partners after having seen them for just a few minutes. Imprinting enables chicks to recognize their mother hen, or any imprinted object, from novel points of view without explicit reinforcement^23^ so that they benefit from protection^24^^,^^25^^,^^26^ and parental care.^27^^,^^28^ Human infants are also capable of rapid generalization from sparse evidence. For instance, 7-month infants can generalize to novel “XXY” vs. “XYX” acoustic patterns, where Xs indicate identical syllables.^29^ A bit later, toddlers shown a single picture of a “rhinoceros” can recognize a different rhinoceros on the television or at the zoo.^30^ Similarly, in their first hours after hatching, chicks^31^ and mallard ducklings^32^ use imprinting to learn the underlying structure of XX (same) vs. XY (different) patterns, and generalize their affiliative responses to entirely novel XX or XY colors and sounds. Precocial birds are therefore ideal models to study spontaneous generalization. Here, we focus on color generalization in poultry chicks (*Gallus gallus*).

Young chicks discriminate color well.^33^^,^^34^^,^^35^ Chickens use color in feeding^36^ and social interactions,^37^^,^^38^ including imprinting.^19^^,^^20^ In imprinting, color is crucial in determining approach responses.^4^^,^^39^^,^^40^^,^^41^ Color generalization in imprinting can be controlled using visual displays.^4^^,^^23^ Here, we report three sets of experiments on imprinting, generalization and spontaneous preferences for colors: Experiments 1–2 compare observed generalization behavior to the predictions of an unbiased model after a short imprinting exposure (1-day); Experiment 3 identifies the spontaneous color preferences present at hatching that might underlie the biases revealed in experiments 1–2; Experiment 4 then examines the time course of learning and generalization, looking at the effects of the biases on longer term learning (5-day imprinting exposure). We then show that spontaneous generalization can be modeled as a Bayesian process, detailing how spontaneous biases (predispositions) drive learning and generalization from sparse evidence in the neonate brain.

### Spontaneous color generalization

**Figure 1:**
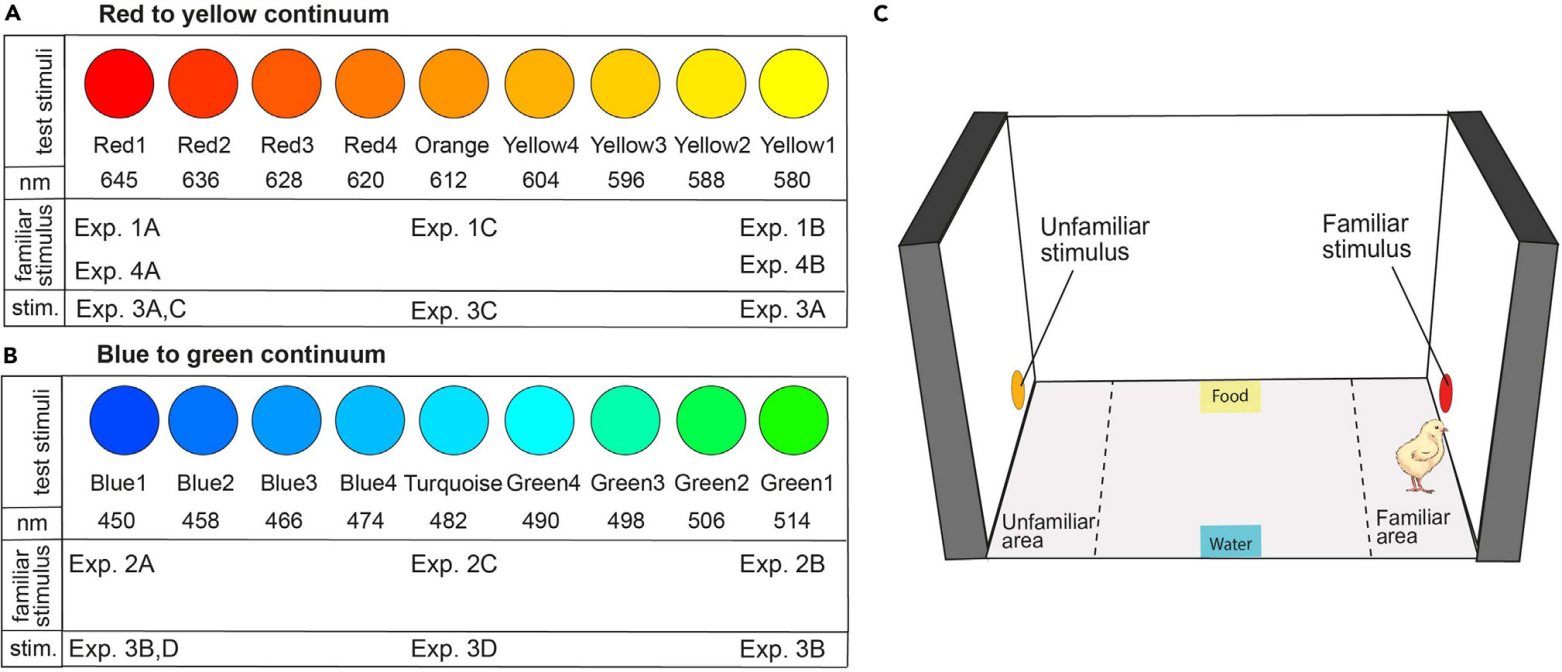
The color continuums of stimuli displayed to chicks and the arena in which experiments took place (A) Red to yellow continuum stimuli. Color, stimulus name and approximate wavelength (to human eye) are reported for each stimulus and experiment. Below, the experiments are listed that use a particular color for imprinting stimulus (“familiar stimulus”, in Exp. 1, 4) or as a choice in predisposition experiments (“stim.” for stimuli in Exp. 3AC). (B) Blue to green continuum stimuli. Color, stimulus name and approximate wavelength are reported for each stimulus and experiment. Below, the experiments are listed that use a particular color for imprinting stimulus (“familiar stimulus”, in Exp. 1, 4) or as a choice in predisposition experiments (“stim” for stimuli in Exp. 3BD). (C) Controlled-rearing apparatus. Dashed lines divide the arena into three regions: familiar stimulus area, center, unfamiliar stimulus area.

We investigated spontaneous generalization using an early exposure and generalization test. Visually naive chicks were first exposed (imprinted) on visual stimuli along either red-yellow (Figure 1A) or blue-green (Figure 1B) color continuums, and then tested for their approach response to the familiar stimulus vs. unfamiliar stimuli on the same continuum.

After hatching in darkness, each chick was housed in an individual home cage (Figure 1C) and exposed to a colored imprinting stimulus for one day (a ∅ 5 cm circle presented for 16 h): either a red (Exp. 1A), yellow (Exp. 1B), or orange (Exp. 1C) circle on the red-yellow continuum; either a blue (Exp. 2A), green (Exp. 2B), or turquoise (Exp. 2C) circle on the blue to green continuum. The imprinting stimulus moved horizontally, switching between the right and left monitor, while the chick moved freely in the arena (see Video S1). A generalization test followed with two different stimuli simultaneously presented (see Video S1); chicks remained in their cage and were offered the choice between a series of pairs of stimuli presented on opposite monitors (12 h), while we measured the preference to approach the familiar stimulus (Figures 1A and 1B) (see Materials and Methods for details). Trials where only the familiar stimulus was presented were used as a baseline of the strength of interest for the imprinting stimulus. The preference for the familiar stimulus was measured as:

$${pref}_{familiar}=\frac{{time}_{familiar}}{{time}_{familiar}+{time}_{unfamiliar}}*100$$

where 100 indicates a full preference for the familiar stimulus, 0 a full preference for the unfamiliar stimulus, and 50% no preference (see also Lemaire et al. and Versace et al.^4^^,^^41^).

**Figure 2:**
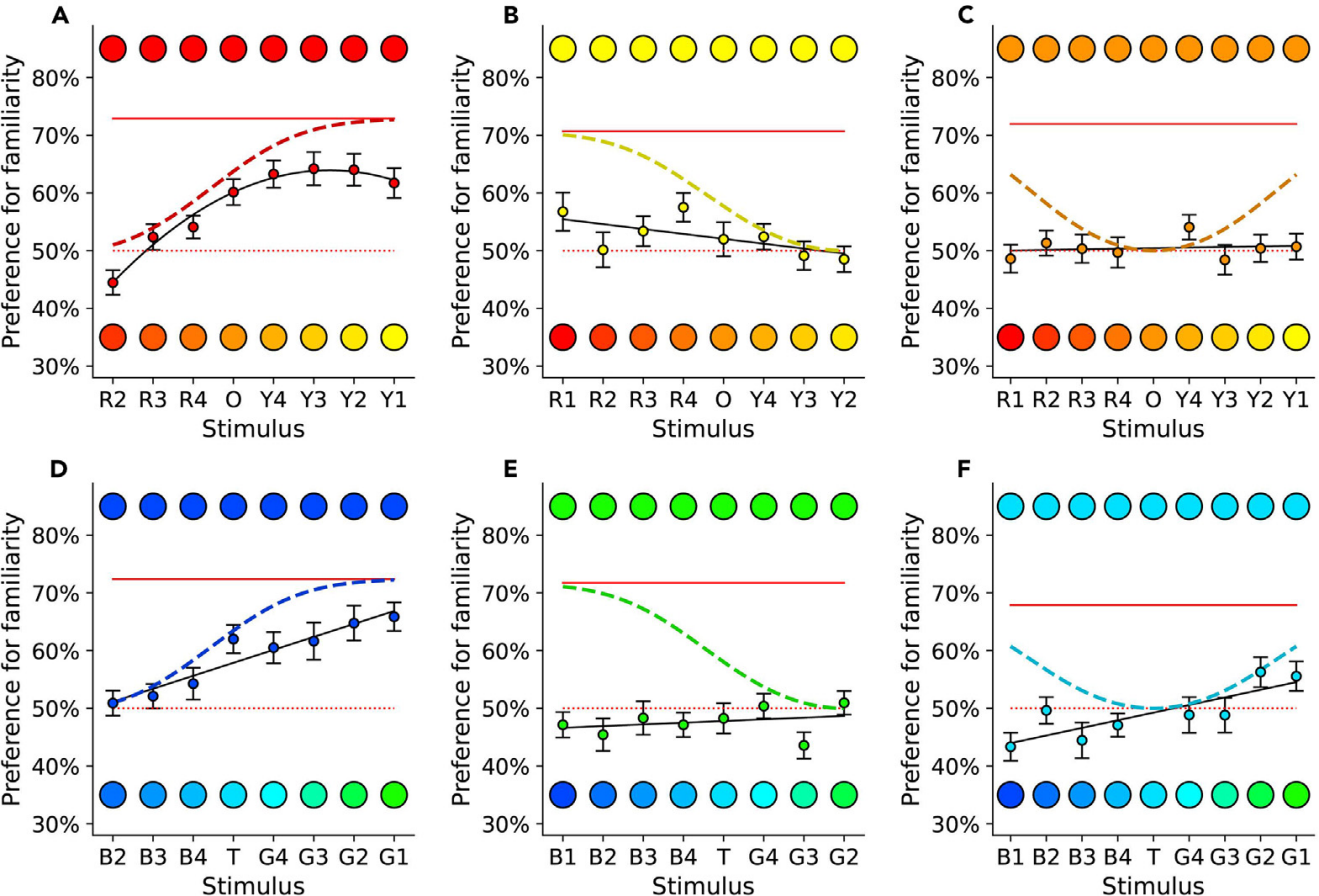
Expected and observed generalization curves For each imprinting stimulus ([A] = Red1, [B]=Yellow1, [C]=Orange, [D] = Blue1, [E] = Green1, [F] = Turquoise), the predicted model for unbiased generalization after one day of imprinting is shown with a dashed line, the observed generalization best fit model as a solid line, with observed data as colored dots +/− SEM. The preference for the familiar stimulus presented alone (rehearsal trials) is a red solid line, while the dotted red line indicates the absence of preference level (50%).

For each imprinting experiment, we compared the experimentally observed generalization curve with the predictions of an unbiased model,^18^ derived from Shepard’s universal law of generalization,^15^ as shown in Figure 2. During imprinting, chicks learn the features of the imprinted object and become attached to it; they then compare it to new objects encountered, to modulate affiliative approach responses based on the perceived similarity. In this unbiased scenario, the similarity between familiar and novel stimuli is dependent solely on direct experience of the colors (modulated by distance in the chicks’ color space, scaled for discriminability).

The unbiased model predicts a monotonic generalization curve (with same order of stimuli in the physiological and perceptual space for humans and birds), where the preference for the familiar stimulus decreases with the distance from this known stimulus^15^^,^^16^^,^^42^ (irrespective to whether we use an exponential^15^ or a Gaussian curve^17^^,^^18^). Although the physical and perceptual distance between stimuli along our one-dimensional color spectrum might be different, their order is maintained in both dimensions, and the distance between two objects along the color continuum is the same in both directions (the distance between Red1 and Red2 is the same as between Red2 and Red1). Unbiased animals are expected to display a progressive decrease of preference for more distant colors. Overall, the unbiased model predicts a consistent preference for the imprinting stimulus (no preference for the unfamiliar stimuli), a peak of preference for the imprinting stimulus, and a progressively decreasing generalization around both sides of the imprinting stimulus (Figure 2).

Some of the observed spontaneous generalization curves violated the predictions of the unbiased generalization model (Figure 2). In Exp. 1, chicks imprinted on Red1 showed a pattern of generalization that followed a quadratic curve (best fit), with increasing preference for familiarity as the distance between the familiar and unfamiliar stimuli increased, and a decrease in preference when the familiar stimulus was matched with the most distant color (Yellow 1) (see Tables S1 and S2). In comparing the preference to chance level, chicks had a significant preference for Red2 (t_45_ = −2.59, *p* = 0.013), and for the familiar Red1 stimulus compared to stimuli along the continuum from Red4 to Yellow1 (Red4: t_45_ = 2.08, *p* = 0.044; O to Y1: t_45_ > 4.53, *p* < 0.001). Conversely, chicks imprinted on Yellow1 had significant preferences for the familiar stimuli in only two cases, with an almost flat generalization curve (see Tables S3 S4; Figure 2B; Red4: t_42_ = 2.99, *p* = 0.005; Red1: t_42_ = 2.04, *p* = 0.048).

In contrast to the prediction of the unbiased model, in Exp. 1A chicks preferred the unfamiliar stimulus Red2 over the familiar Red1 stimulus (Figure 2A, t_45_ = −2.59, *p* = 0.013). This preference suggests that the chicks have a predisposition to imprint on a color less extreme than Red1 on the red to yellow continuum.

In Exp. 1C and 2C, we imprinted chicks on intermediate colors Orange and Turquoise, respectively. Chicks imprinted on Turquoise showed significant preferences in three cases (Green1: t_44_ = 2.17, *p* = 0.035, Green2: t_44_ = 2.42, *p* = 0.020, Blue4: t_44_ = -2.74, *p* = 0.009) and exhibited a linear generalization gradient (see Table S9-S12) resembling that observed after imprinting with the color Blue1, but with a lower intercept. Hence, the unbiased model prediction of a central peak and progressive reduction of preference with more distant colors was rejected (Figure 2F). Similarly, chicks imprinted on Orange showed a linear flat gradient (Figure 2C) with no significant preferences, unlike what predicted by the unbiased model.

Importantly, all chicks received the same amount of imprinting exposure in Exp. 1 ABC and Exp. 2 ABC, they were equally engaged during the imprinting phase (no significant difference in preference between any of the colors used, *F*_5, 295_ = 0.811, *p* = 0.543), and had similar baseline preferences for the imprinting stimulus presented alone with a blank alternative monitor on the opposite side (Figure 1; Red1 = 73%, Yellow1 = 71%, Blue1 = 72%, Green1 = 72%, Orange = 72%, Turquoise = 68%). Therefore, the differences in generalization between Red1 vs. Yellow1 and Blue1 vs. Green1 imprinted chicks are not due to different salience of the stimuli (red solid lines in Figure 2). This shows that from the beginning of life, in the absence of previous experience, equal exposure to different stimuli along a color continuum can have dramatically different outcomes in generalization.

Overall, the results reject the unbiased generalization model, indicating that spontaneous biases (biases not dependent on reinforcement or previous experience) shape generalization in the neonate brain. Subsequent experiments assessed whether biases in learning and generalization correspond to early spontaneous preferences to approach a particular color (Exp. 3, section Colour predispositions and imprinting generalization), and the consequences of predispositions for the time course of learning and generalization (Exp. 4, sections Time course of generalization and Conditional probabilities drive the evolution of predispositions).

### Color predispositions and imprinting generalization

Soon after birth, infants and domestic chicks show spontaneous preferences (predispositions,^7^ see Box 1) for certain stimuli, including particular colors (see Table S14), moving objects,^43^^,^^44^^,^^45^ stuffed hens,^46^^,^^47^ hollow objects,^41^ face-like stimuli,^48^ or combinations of features such as color and biological motion.^49^ Lesion experiments have shown a dissociation between brain areas involved in predispositions and learning.^8^^,^^50^ Whether and how predispositions drive learning and generalization at the beginning of life is unclear. While previous work (see in the study by Zylinski et al. and Gittleman et al.^10^^,^^51^ and references therein) has shown that colorful aposematic signals facilitate predators in learning what prey is unpalatable, and that biases affect the rate of conditioned learning,^11^^,^^12^^,^^13^ the principles of spontaneous (not reinforced, unsupervised) and early generalization are unknown. We tested whether predispositions affect unsupervised learning and generalization in inexperienced animals. By focusing on spontaneous generalization, we model the many settings when animals cannot rely on repeated trial and learning, and nonetheless succeed in inductive generalization.

Box 1 **Predispositions**

Predispositions are early life biases that, before experience takes place, influence the behavior of animals, direct attention, and approach/avoidance responses toward particular objects.^38^^,^^39^^,^^40^ Neonate vertebrates seem to be predisposed for animacy cues, namely features associated with the presence of living beings.^38^^,^^39^^,^^40^^,^^45^ Predispositions orient the attention of inexperienced young animals toward specific stimuli, but it remains an open question whether predispositions influence learning and generalization.^29^ In chicks, several predispositions to preferentially orient toward and approach some stimuli have been identified.^38^^,^^39^ Initial preferences for moving objects have been long known, and recent studies have identified specific features of moving objects that elicit approach responses: changes in speed,^41^^,^^42^ self-propulsion,^43^ movement against gravity,^44^ and biological motion.^46^ Based on the central role of moving stimuli, we imprinted chicks on moving objects. Predispositions are present also for static features, such as particular colors,^4^^,^^47^^,^^48^^,^^49^ for stuffed hens,^37^^,^^50^ hollow objects,^24^ face-like stimuli,^51^ and for a combination of features such as color and biological motion.^52^ Human babies^52^^,^^53^^,^^54^ and even tortoises^55^ have similar predispositions, suggesting that they are of broad evolutionary significance. Interestingly, the neural substrates of predispositions are at least partially distinct from those of learning mechanisms such as imprinting,^8^^,^^56^ implying that there is a separation between the initial orienting response to “salient” objects and imprinting responses. Here, we investigate the connection between predispositions and generalization of color in filial imprinting.

**Figure 3:**
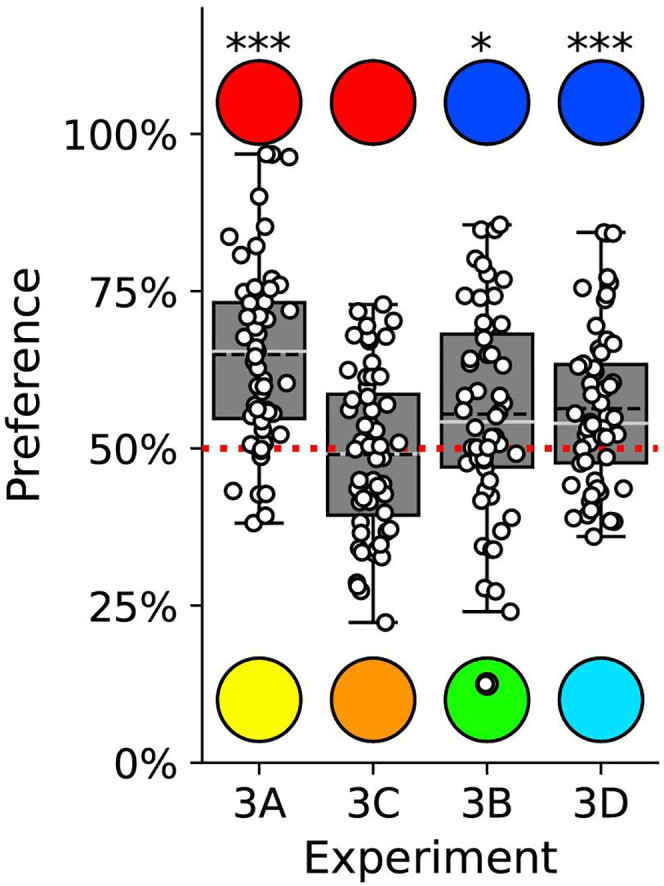
Average preference indices in the experiments 3A-3D, calculated for the predisposed Red1 and Blue1 stimuli Boxplots display mean (black dashed line), median (white line), quartiles and outliers. Red dashed lines represent no preference (chance level) in a one-sample t test; ∗*p* < 0.05; ∗∗*p* < 0.01; ∗∗∗*p* < 0.001.

Experiment 3 assessed color predispositions for the colors tested in Exps. 1 and 2. If color predispositions drive generalization, inexperienced chicks should prefer Red1 over Yellow1, and Blue1 over Green1, because these colors elicited fast learning and a clear generalization gradient after imprinting. We therefore recorded color preferences in a double choice test in the first 2 h of visual exposure,^57^ at the start of the imprinting process. Exp. 3A tested Red1 vs. Yellow1, and Exp. 3B Blue1 vs. Green1. As predicted, chicks preferred Red1 over Yellow1 (mean ± SEM = 0.649 ± 0.018, t_58_ = 8.12, *p* < 0.001, Figure 3) and Blue1 over Green1 (mean ± SEM = 0.554 ± 0.022, t_53_ = 2.40, *p* < 0.020, Figure 3). These preferences match the robust gradients of generalization for chicks imprinted on Red1 and Blue1 that were observed in Exp. 1 and 2, confirming a potential role of early predispositions in generalization.

We also assessed the preference for the intermediate stimulus vs. the preferred stimulus (Orange vs. Red1: Exp. 3C, and Turquoise vs. Blue1: Exp. 3D), aiming to understand the shape of the initial preference curve. In Exp. 3C, chicks showed no preference for Red1 vs. Orange (mean ± SEM = 0.490 ± 0.017, *t*_55_ = −0.58, *p* = 0.565, and in Exp. 3D chicks preferred Blue1 over Turquoise (mean ± SEM = 0.563 ± 0.017, *t*_53=_3.66, *p* < 0.001, Figure 3). The preference for Blue1 over Turquoise suggests that the linear generalization observed in Exp. 2C reflects a predisposition for Blue1, while the lack of preference between Red1 and Orange might explain why, in Exp. 1C, imprinting exposure on the intermediate stimulus (Orange) didn’t produce a generalization gradient.

Overall, the correspondence between fast learning and generalization after imprinting with some colors (Red1 and Blue1) but not others (Yellow1, Green1, and Orange), with early spontaneous preferences for the same colors, and the bias in generalization (after imprinting with Turquoise), show that neonate chicks are evolutionary prepared for generalization, via biases that do not depend on reinforcement and supervision.

### Time course of generalization

To investigate how predispositions affect the time courses of imprinting and generalization, we focused on the red to yellow continuum. Based on previous evidence (Exp. 1–3), we hypothesized that differences in generalization curves are attributable to the chicks’ early color predispositions. Accordingly, we used a longer (5-day) imprinting experiment on either Red1 or Yellow1 (Exp. 4A and B) to test the predictions of a theoretical model that explicitly incorporates predispositions as initial preferences and treats the imprinting process as an update of these preferences.

The model assumes that each chick has an underlying preference for each stimulus and chooses between them in proportion to their evaluated value:

$${choice}_{i,j}=\frac{{pref}_{i}}{{pref}_{i}+{pref}_{j}}$$

where ${pref}_{i}$ and ${pref}_{j}$ represent the value the chick assigns to the stimulus, and ${choice}_{i,j}$ indicates the proportion of choices of stimulus i over stimulus j. When comparing the model predictions to empirical observations, we scaled the model predictions to the maximum observed preference (either in rehearsal trials or during the tests).

During imprinting, chicks use their experience to re-evaluate and adjust their preferences. The learning process is represented here as a Gaussian and multiplicative update of the preferences. At each time step t, preferences across the stimulus continuum are updated as:

$${pref}_{i,t+1}={pref}_{i,t}*\left(1+\alpha *w_{i,t}\right)$$

where the update rate α is a constant that defines the speed of learning, and the update weight function w describes the experience of the chick. It is maximal at the stimulus value presented at time t, and has a narrow Gaussian distribution around the peak. Preferences for stimuli that are perceptually very similar to the exemplar are updated as well. At each time step, the preference curve is normalized to its maximum.

**Figure 4:**
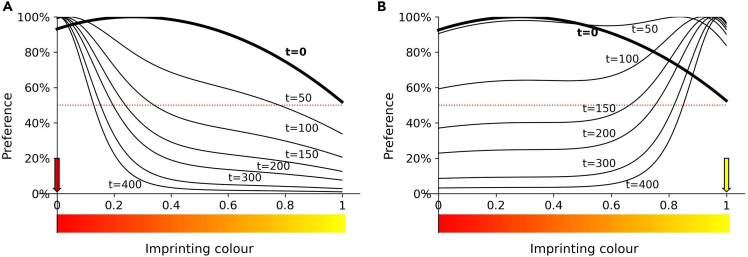
Predicted color preferences over time The preferences develop differently when imprinting on Red1 (A) and Yellow1 (B), despite converging onto the same strong preference for the imprinting stimulus. The red and yellow arrows highlight the imprinting stimulus, and “t” indicates time step.

We applied this model to the red-yellow continuum (Figure 4), using the initial preference curves estimated from Exps. 3A,C, where Red1 and Orange were equally preferred at hatching, and approximately twice as attractive as Yellow1. In choosing Red1 and Yellow1 as the imprinting stimuli, we iterated the update process for 400 time steps, setting α=0.01 and the update curve’s standard deviation to σ=0.2, and calculated the preference curves at each time step. The model predicts that preferences for the predisposed Red1 and the non-predisposed Yellow1 develop differently (Figure 4). By the end of the simulation, the imprinting stimulus is strongly preferred for in both cases, but the preference curves differ at intermediate time steps. For the predisposed Red1, the preference forms quickly, and the imprinting narrows it over time. In contrast, for the non-predisposed Yellow1, establishing the preference takes a longer (imprinting) time, with an intermediate phase when all stimuli on the red-yellow continuum are expected to be equally attractive.

Experiment 4 tested the model empirically on the red-yellow continuum, comparing its predictions to the results of the 1-day imprinting experiments (Exp. 1A, 1B) and running a new, longer experiment, where generalization is tested after five days of imprinting, when the learning is completed and stable.^5^ We used the same imprinting procedure, generalization test and analyses as for the 1-day imprinting experiments (Exp. 1 and 2), the only difference being that the imprinting exposure was repeated for five consecutive days before the generalization test.

**Figure 5:**
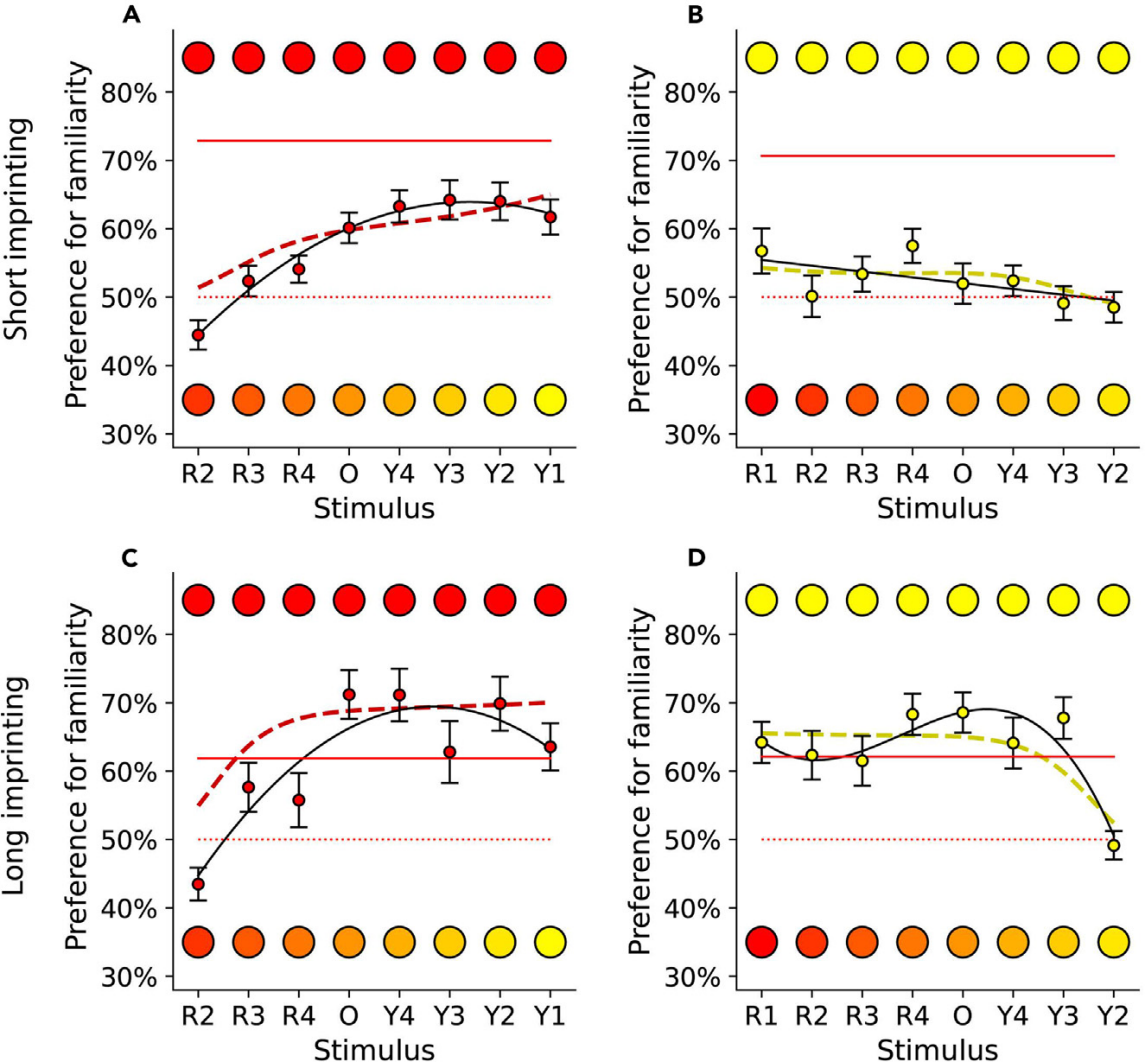
Expected and observed generalization curves for short and long imprinting Experimentally observed vs. predicted generalization curves after short (1-day; t = 100) and long (5-day; t = 300) imprinting experience, for Red1 (A and C) and Yellow1 (B and D) imprinting stimuli (Exps. 1, 4). For each imprinting stimulus and duration of imprinting, model predictions are shown with a dashed red line, the observed generalization best fit model as a solid line, and the observed data as colored dots +/− SEM. The preference for the familiar stimulus presented alone (rehearsal trials during Exps. 1,4) is a red solid line, while the dotted red line indicates the absence of preference. The predictions of the theoretical model match the empirical observations. Note that in the long experiments (Exp. 4), the rehearsal trial preference is lower than preference in the dual choice experiments (after the end of the sensitive period, chicks actively avoid the unfamiliar stimulus when an option is given).

We found that the model reflects the results of the short 1-day imprinting (Exp. 1A, 1B) and the long 5-day imprinting experiments (Exp. 4A, 4B) (Figure 5). For the short (one day) imprinting experiments, the model predicts a gradually increasing preference for the familiar stimulus when imprinted on Red1 (Figure 5A) and a slight preference for Yellow1 over unfamiliar stimuli. After the long (5 days) imprinting (Exp. 4), the predicted generalization curves have similar shapes for both imprinting colors: generalization to the most similar color, and then a sharp increase of preference to a plateau. The model captures most of the variation in the experimentally observed generalization curves: Exp. 1A, short imprinting on Red1: R^2^ = 0.979; Exp. 4A, long imprinting on Red1: R^2^ = 0.807; Exp. 4B, long imprinting on Yellow1 R^2^ = 0.738. For Exp. 1B, short imprinting on Yellow 1, the trend in the observations is small and accordingly the R^2^ value is low, 0.392. We conclude that accounting for the initial preferences, and treating the imprinting as an update of these initial preferences, is sufficient to explain the different generalization curves and time courses for different colors. This conclusion underlines the importance of predispositions in learning from the first day of life, in the absence of any reinforcement and previous experience.

### Conditional probabilities drive the evolution of predispositions

It is not clear whether a chick that has to recognize the imprinting target in a generalization task (e.g., recognizing the mother hen from a novel point of view or when her feathers are located in a novel configuration, or choosing the mother when multiple hens are present) can find the task easier by focusing on specific features, such as color. As we review in Table S14, several studies^58^^,^^59^^,^^60^^,^^61^^,^^62^^,^^63^^,^^64^^,^^65^^,^^66^^,^^67^ have focused on the similarity between predispositions for specific features (e.g., a particular color^58^) and the characteristics of the mother hen. However, preferences for different colors vary between experimental settings, suggesting that the physical characteristics of the stimuli (e.g., a specific wavelength) are not sufficient to capture observed behavior. We use a different approach, focusing on the relative preferences, considering the task similar to a likelihood test. We formalize this idea modeling the chick as an ideal Bayesian observer that evaluates each potential target according to the conditional probabilities calculated with Bayes’ theorem:

$$P\left(target|colour\right)=\frac{P\left(colour|target\right)*P\left(target\right)}{P\left(colour|target\right)*P\left(target\right)+P\left(colour|not\_target\right)*P\left(not\_target\right)}$$

where *P*(*target*|*color*) is the probability that the chick assigns to the hypotheses that an object seen is the target (the mother hen), given its observed color. In our formulation we assume that the chick hatches with a set of expectations (predispositions), both about the probability distribution of the features of the ideal target *P*(*color*|*target*), and about the probability distribution of the features of other objects *P*(*color*|*not_target*). We will treat the probability that any given object is the target *P*(*target*) or something else *P*(*not-target)* as constants. It follows that the likelihood ratio of an object with a given color being the target, and therefore the interest the chick should show in it, will increase with the probability that the target displays that particular color *P*(co*lor*|target), and decrease with the prevalence of the color in other objects present in that environment *P*(*color*|*not_target*).

**Figure 6:**
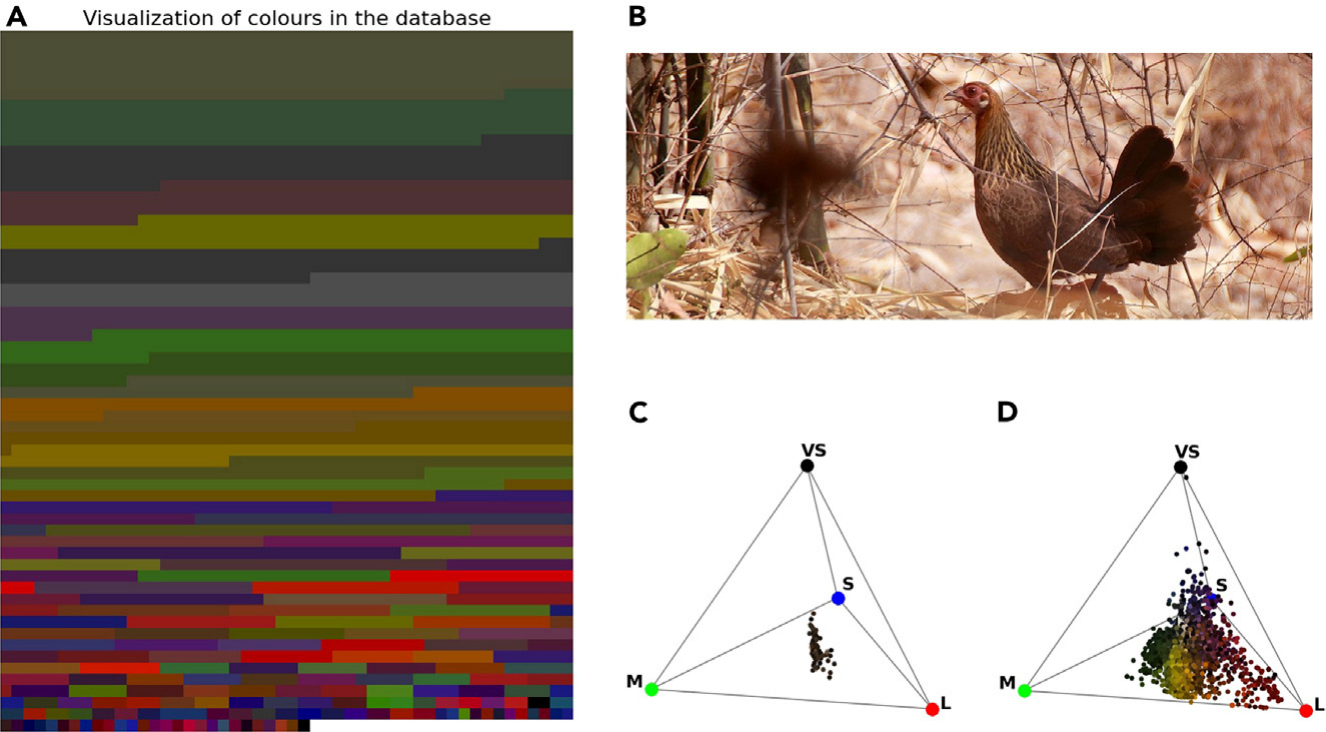
Natural colors and red jungle fowl colors (A) We calculated the red, green and blue photoreceptor responses of a typical UV-sensitive bird^68^ to a large library of natural colors^69^^,^^70^ and visualized them as RGB values. The area of each colored stripe corresponds to its frequency in the database. Natural objects—flowers, leaves, rocks, soil—appear as unsaturated greens, browns and greys to the birds. Red colored objects exist, but they are rare. Note that the colors have been normalized for equal total brightness, and that the visualization ignores the UV dimension of color. (B) The red junglefowl hen (the closest relative of the domestic chicken) is predominantly yellow/brown, with reddish areas in the head region. Photo credit: Brian Gratwicke, downloaded from https://commons.wikimedia.org/wiki/File:Red_Junglefowl_hen_India.jpg. (C) The color loci of the red junglefowl hen feathers ^58^ and of (D) natural objects, plotted in the tetrahedral color space of birds. The tips of the tetrahedron correspond to situations where only one receptor is activated. VS—UV/violet sensitive receptor, S—short wavelength (blue) sensitive receptor, M—medium wavelength (green) sensitive receptor, L—long wavelength (red) sensitive receptor.

From an evolutionary point of view, the ideal imprinting targets for non-domesticated chicks, the mother hens, display yellow and brown colors with reddish areas in the head region (Figure 6B^58^). In a previous study, we investigated the feather and head colors of red junglefowl hens (the wild species closest to the ancestral domestic chickens), using spectral reflectance measurements of bird skins.^58^ The feathers’ reflectance spectra increases with wavelengths (characteristic of phaeomelanin), giving a yellowish-brown appearance. Bird single cone photoreceptors’ responses to these spectra are plotted in a tetrahedral color space (Figure 6C). The feather colors of various areas of red junglefowl hens fall close the achromatic middle point, except for the lack of UV. In addition to the feathers, the soft parts of the head regions (comb, eye) appear reddish.^58^ Hence, red junglefowl hens display both reddish and yellowish hues, and the coloration of the mother hens does not explain the chicks’ preference for red in itself.

Turning to the colors of the natural environment, we used spectral reflectance functions of a variety of materials including soil, minerals, vegetation, and flowers, available in public databases (FReD^69^ and USGS^70^; see Methods). Plotting the avian photoreceptor responses^68^^,^^71^ in tetrahedral space,^68^ reveals a lack of saturated colors, and that red is rare while greens/browns/dark yellows dominate (Figure 6). Against such a background, red junglefowl plumage colors would be hard to identify, but the reds of the soft parts on the head would stand out. Therefore, as there is a larger probability that a hen (target) displays a red color *P*(*color***|***target*) (even though it is primarily brown), and a smaller probability that a non-target object in the environment is red *P*(*color***|***not_target*), we conclude that the color red is a good indicator for identifying the target mother hen *P*(*target***|***color*). For this reason, red (and not yellow) color can be used by newly hatched chicks initially for orienting toward the mother hen, and then recognize the target compared to alternative objects, making red color an optimal target for early predispositions. Therefore, Bayesian conditional probabilities offer an explanation for the red over yellow preference we have observed in Exp. 1, 3, and 4. Similarly, the lower conditional probabilities of blue compared to green in the natural environment could explain the stronger preference for blue over green and turquoise observed in Exp. 2 and 3.

#### General discussion: Rara avis in terris

To cope with environmental changes and novelty, it is necessary to go beyond previously observed data and solve the *problem of induction*, making connections between previous observations and expectations or predictions about what has not yet been observed.^72^ In recent decades it has emerged that brains work by making predictions, rather than simply by reacting to sensory stimuli.^73^ After having seen only white swans, one might predict that the next swan will be white. This reasoning does not apply only to birds. The Roman poet Juvenal, almost two thousand years ago, referred to a *rara avis in terris*, *nigroque simillima cygno*, “a bird as rare as a black swan”, to indicate an exceptional person, with features that are not expected or predicted. But where do expectations come from? Generalization is especially demanding when experience is limited, as at the beginning of life or in any new context. Our research focused on unsupervised generalization, in the absence of trial and learning opportunity, as we encounter in novel contexts in our everyday life. Differently from current artificial intelligence systems,^2^ in many domains infants,^29^ children,^30^ and other young animals^31^ generalize effectively from sparse evidence, but the strategies and mechanisms that they use are unknown. This knowledge gap derives from the difficulties of testing naive individuals whose experience is controlled from birth to test. Ethical and practical issues prevent and limit controlled-rearing studies in human babies and altricial species. To address this issue, we investigated the principles of spontaneous generalization in neonate and visually inexperienced chicks, which are precocial animals hatched with a mature sensory and motor system. Chicks were controlled-reared from hatching until the moment of test in an arena with colored displays. We investigated learned and spontaneous approach responses to colored moving objects, which young chicks treat as social partners, and compared empirically observed generalization (choice between a familiar and several unfamiliar shapes with colors progressively more distant from the familiar ones) with the predictions of an unbiased generalization model.^61^

The results reject the unbiased model,^15^ implying that the predispositions^7^ that orient first approach responses facilitate inductive generalization in neonate chicks. First, inconsistent with unbiased predictions, chicks exposed and then tested for generalization on the red to yellow and blue to green continua exhibited completely different generalization curves, depending on their imprinting experience (Red1, Yellow1, Orange or Blue1, Green1, Turquoise in Exp. 1ABC, Exp. 2ABC, respectively). Chicks had been exposed to each imprinting stimulus for the same amount of time, and were equally responsive to each stimulus during imprinting. Despite this, only those imprinted on Red1 and Blue1 exhibited a robust generalization gradient, while chicks imprinted on Yellow1, Orange, and Green1 showed no preference for either the familiar or unfamiliar stimuli, and chicks imprinted on Turquoise did not show the expected peak preference for the familiar stimulus. This is revealing, given that the same stimuli (with the same discriminability) have been used in the red-yellow/yellow-red and in the blue-green/green-blue continuums. These differences in generalization after similar experience are incompatible with an unbiased model and point toward an evolutionarily prepared model of generalization, with some expectations that drive inductive generalization in place already at birth, before visual experience.

We asked whether learning and generalization to novel objects are driven by early predispositions to approach different colors. Predispositions to approach different colors in the first visual experience (Exp. 3ABCD) matched the results of the generalization test: chicks spontaneously preferred Red1 over Yellow1, Blue1 over Green1, and Blue1 over Turquoise, but had no preference for Red1 vs. Orange. This corresponds to a scenario where neonate chicks operate as Bayesian observers.^42^ In fact, red and blue colors are disproportionately present in objects that are likely to be the imprinting target, rather than in background objects, as shown by our analysis of fowl and environmental colors^58^^,^^69^^,^^70^ (section Conditional probabilities drive the evolution of predispositions). A further prediction derived from this model is that red should be preferred over brown, even though brown is more dominant in the natural hen coloration.

Second, we observed preferences for the unfamiliar stimulus Red2 (Exp. 1 and 4), whereas the unbiased generalization model^28^^,^^31^ predicts a preference for the imprinting object (Red1). A preference for unfamiliar stimuli^31^ or for stimuli “slightly different”^57^ from the imprinting object has been repeatedly observed in chicks. The preference for unfamiliar stimuli was initially observed at the beginning of the imprinting process,^57^ and described as the outcome of two complementary components: the attraction for familiar objects and that for novel objects. This dual process would allow young birds to gain as much information as possible about their specific mother and siblings from the different view-points, to enhance subsequent recognition. However, this explanation cannot account for why preferences for unfamiliar objects have been observed also in older chicks, after several days of imprinting exposure,^7^^,^^31^^,^^74^ and the selective presence of novelty preferences that we observed only for certain novel objects. This pattern suggests that the preference for unfamiliar stimuli might be due to initial biases for specific colors, and in particular that Red1 is not the most initially preferred color for chicks in the Red1 to Yellow1 continuum. This is supported by the observation that Red1 and Orange were equally attractive to naive chicks (Exp. 3C). The preference for colors different from the imprinting stimulus observed after one day, and even five days of imprinting, supports the hypothesis that spontaneous, naturally selected biases influence chicks’ learning and generalization: they have prepared minds. These biases are likely to reflect the evolutionary significance of particular colors. A predisposition to approach red stimuli that could be conspecifics has a selective advantage (see Gamberale-Stille et al.^75^ for food items), because the head region of the red jungle fowl hens, the closest relatives to ancestral chickens before domestication, is reddish.^58^^,^^75^ Hence, a predisposed preference for red could help chicks to selectively process and learn from reddish stimuli, that are associated with the target imprinting stimulus.

Overall, we show how spontaneous biases (predispositions) for behaviorally relevant stimuli can facilitate learning with limited experience, leading to faster and more robust generalization, compared to generalization from less predisposed stimuli. This finding has broad implications for understanding the development of cognition in biological and artificial minds. Predispositions not only guide early orienting approach responses but they directly affect learning and generalization, showing that the neonate brain makes predictions consistent with Bayesian models, in the absence of previous experience and reinforcement.

Moreover, the connection between early predispositions and generalization suggests that low level biases or Bayesian expectations can be implemented as building blocks of more sophisticated cognitive abilities, with the potential to improve artificial intelligence that currently requires extensive training or preparation.^2^ AI may benefit from priors or biases similar to those used by infants, to more efficiently process and learn evolutionarily- or task-significant information. By identifying the biases that influence an animal’s learning, researchers may be able to design systems that can quickly adapt to novel environments, and to understand the building blocks of cognition.

#### Limitations of the study

This study investigates the presence and effect of biases in spontaneous early preferences and imprinting responses to visual objects in poultry chicks. Due to practical limitations, we focused only on some colors along the red to yellow and blue to green continuums. We used RGB/luminance values previously used in other studies. Future studies should expand to different colors, luminance values and species. Due to technical limitations of RBG monitors, the colors seen by chicks varied depending in the exact position of the chicks.

## STAR★Methods

### Key resources table

**Table:** 

REAGENT or RESOURCE	SOURCE	IDENTIFIER
**Deposited data**		

Dataset Exp. 1	This paper	https://doi.org/10.5281/zenodo.10869154
Dataset Exp. 2	This paper	https://doi.org/10.5281/zenodo.10869154
Dataset Exp. 3	This paper	https://doi.org/10.5281/zenodo.10869154
Dataset Exp. 4	This paper	https://doi.org/10.5281/zenodo.10869154

**Experimental models: Organisms/strains**		

Domestic chicken (*Gallus gallus*)	PD Hook, Cote, Bampton, UK	Strain: Ross 308

**Software and algorithms**		

**DeepLabCut**	Mathis, A., Mamidanna, P., Cury, K.M., Abe, T., Murthy, V.N., Mathis, M.W., and Bethge, M. (2018). DeepLabCut: markerless pose estimation of user-defined body parts with deep learning. Nature Neuroscience *21*, 1281–1289. 10.1038/s41593-018-0209-y.	https://www.nature.com/articles/s41593-018-0209-y
**Code**	This paper	https://doi.org/10.5281/zenodo.10869154

### Resource availability

#### Lead contact

Further information and requests for resources should be directed to and will be fulfilled by the lead contact, Elisabetta Versace (e.versace@qmul.ac.uk).

#### Materials availability

This study did not generate new unique reagents.

#### Data and code availability

• Behavioural data have been deposited on Zenodo at https://doi.org/10.5281/zenodo.10869154. They are publicly available as of the date of publication and DOIs are listed in the key resources table.
• Original code has been deposited on Zenodo at https://doi.org/10.5281/zenodo.10869154 and is publicly available as of the date of publication. DOIs are listed in the key resources table.
• Any additional information required to reanalyze the data reported in this paper is available from lead contact upon request or from staff mentioned in Zenodo’s files.

### Experimental model and study participant details

#### Methods details

##### Ethics

Experiments complied with national laws on the use of‬ animals in research and were approved by the Home Office (PPL number: PP5180959) and by the Queen Mary University of London Animal Welfare and Ethical Review Body‬‬‬‬‬‬‬‬‬

#### Imprinting: Experiment 1,2 and 4

##### Subjects

We tested the following number of chicks: Exp. 1A: 24 males, 24 females; Exp. 1B: 24 males, 24 females; Exp. 1C 28 males, 31 females; Exp. 2A: 24 males, 24 females; Exp. 2B: 24 males, 24 females; Exp. 2C 26 males, 24 females; Exp. 4A 15 males, 13 females; Exp. 4B: 15 males, 20 females. All chicks were tested within the first 24 hours after hatching. Chicks were randomly assigned to the different conditions/experiments. Power analysis run prior to the experiment indicated for the imprinting experiments a sample size of 48 animals for a medium effect size d = 0.4, with alpha = 0.05 and power = 0.8.

#### Colour predispositions: Experiment 3

##### Subjects

In Experiment 3, we used 223 domestic chicks: Exp. 3A: 34 males, 25 females; Exp. 3B: 25 males, 29 females; Exp. 3C 25 males, 31 females; Exp. 3D: 23 males, 31 females. Chicks were within 24 hours after hatching at the beginning of the experiment. Power analysis run prior to the experiment indicated for the imprinting experiments a sample size of 60 for a medium effect size d = 0.37 with alpha = 0.05 and power = 0.8. Chicks were randomly assigned to the different conditions/experiments.

#### Imprinting: Experiment 1,2 and 4

##### ‬‬‬‬‬‬‬‬‬‬‬‬‬‬‬‬‬‬‬‬‬‬‬‬‬‬‬‬‬‬‬‬‬‬‬‬‬‬‬‬‬‬‬‬‬‬‬‬‬‬‬‬‬‬‬‬‬‬‬‬‬‬‬‬‬‬‬Incubation and rearing conditions

Fresh eggs of domestic chicks (*Gallus gallus*) Ross 308 were used in all experiments (from PD Hook, Cote, Bampton, UK). Eggs were incubated and hatched in darkness under controlled conditions (37.7°C and 40% humidity). Chicks hatched in opaque individual boxes, and had no visual experience before the experiment. Within 8-18 hours from hatching, chicks were sexed and individually placed in their home cage with water and food available *ad libitum*. Chicks were exposed to a day:night cycle of 16:8 hours, alternating stimuli (day) and dark slides (night) displayed on the screens.

##### Apparatus

The home cage was used as experimental apparatus. Apparatuses were rectangular (90 x 60 x 60 cm) enclosures, with white walls and a computer located on each of the short sides of the arena (Figure 1C). Stimuli were displayed on high-frequency 24-inch widescreen monitors (ASUS MG248, 144 Hz). Before starting the experiments, we matched the luminance and hue of the monitors. A webcam located above the centre of the arena recorded chicks’ behaviour. Food and water were placed in the middle of the long walls, available *ad libitum*. The areas within 20 cm of each monitor were defined as the regions near familiar/unfamiliar stimulus. These areas were chosen as chicks spent above 90% of their time in these regions (average across all chicks from Exp. 1A).

##### Stimuli

Stimuli were circles of different colour with a diameter of 5 cm, including a 1.5 mm black outline, presented on computer monitors. Previous experiments showed the efficacy of similar visual displays as imprinting objects. The approximate wavelengths for all test stimuli are shown in Figures 1A and 1B, with corresponding RGB values listed in Table S13. We matched visual settings of the monitors and standardised the brightness of the stimuli using a lux meter (AMPROBE LM-120). In each experiment we presented one imprinting stimulus and eight test stimuli. The stimuli move horizontally at a speed of 10 cm/s, with a frame rate of 120 fps. As the stimulus reached one end of the screen, it disappeared out of the frame for 0.5 seconds before reappearing. Videos were displayed using Potplayer (1.7.17508).

##### Procedure

The experiment consisted of two phases: imprinting and test phase. In Exp. 1 and 2, the imprinting phase took place on day 1, in Exp. 4 on day 1-5, During the imprinting phase, chicks were exposed to a single imprinting stimulus that was displayed on one of the screens. The side of the display screen was counterbalanced. Each chick underwent 16 hours of imprinting (8 x 2-hour imprinting sessions). We focused the analyses on the first 12 hours, since in Exp. 1 we noticed that in the last 4 hours chicks spent a high proportion of time asleep. Each imprinting session consisted of 10 trials, with 10 minutes of stimulus presentation in each trial, followed by 2 minutes of empty white screen.

The test phase took place either on day 2 (Exp. 1 and 2) or on day 6 (Exp. 4) after hatching. The test phase consisted of eight sessions of 10 trials (10 minutes of stimulus presentation followed by 2 minutes with a white screen). Each session began with two rehearsal trials which were identical to the imprinting trials (only one imprinting stimulus was presented on one monitor). This was followed by eight test trials, in which chicks were exposed to two stimuli, each presented on a different monitor: the imprinting stimulus and one of the test stimuli. A different test stimulus was presented in each trial. Therefore, each chick experienced all eight test stimuli in one session.

#### Colour predispositions: Experiment 3

##### Stimuli

We used a subset of the stimuli described in 3.2 and illustrated in Fig. 1A. In Exp. 3A we tested the preference for Red1 vs Yellow1, in Exp. 3B we tested the preference for Blue1 vs Green1, in Exp.3C Red 1 vs Orange, in Exp. 3D Blue1 vs Turquoise.

##### Procedure

After hatching in darkness, chicks were sexed and individually placed in their apparatus. Each chick underwent six consecutive trials of double choice test, with one stimulus presented on each screen. Each trial consisted of 20 minutes of stimulus presentation, followed by 2 minutes of white screen, for a total of 120 minutes of stimulus presentation The right-left side on the stimuli was counterbalanced between trials.

### Quantification and statistical analysis

#### Imprinting: Experiment 1,2 and 4

##### Data analysis

Statistical analyses were performed using R (version 4.0.4), packages ply4, lme4, ez, tidyverse, ggplot2, and gridExtra and Google Colab using Python’s scipy and statsmodels packages. Alpha level was set to p≤0.05. P values have been corrected for multiple comparisons using Bonferroni-Holm correction.

The position of chicks’ and stimuli’s centroid during the experiments were analysed using the automated tracking software DeepLabCut.^76^ We analysed only frames tracked with a likelihood reliability of 0.9 or above^77^: in Exp. 1 and 2 this corresponded to 98% of recorded frames, in Exp. 4 to 99% . The preference for the familiar stimulus was measured as:

$${pref}_{familiar}=\frac{{time}_{familiar}}{{time}_{familiar}+{time}_{unfamiliar}}*100$$

where a preference index of 50% indicated no preference for either stimulus, a preference higher than 50% indicated a preference for the imprinting stimulus, and a preference lower than 50% is preference for the novel stimulus (see also^4^^,^^41^).

To assess the chicks’ preference for the imprinting stimulus during the imprinting phase we ran mixed-model ANOVA with preference scores as the dependent variable, Sex (male, female) and Stimulus as the independent variables. One-sample t-tests were used to assess whether chicks spent significantly more time near the imprinting stimulus than chance level (50%). For the test phase, a two-way mixed-model ANOVA was run to assess the effect of Stimulus on chicks’ preferences for familiarity, with the percentage of time spent near the imprinting stimulus as dependent variable and Sex (male, female) and Stimulus as independent variables. Greenhouse-Geisser correction was applied to within-subjects factors that violate the sphericity assumption. We used one-sample t-tests to assess whether chicks spent significant different time near the imprinting stimulus than chance level (50%) when presented with each novel stimulus. Independent samples t-tests were used to assess whether chicks showed significantly different preferences towards the imprinting stimulus when presented with a test colour from the continuum half on either side of the intermediate imprinting stimulus.

#### Colour predispositions: Experiment 3

##### Data analysis

We used two measures of spontaneous preferences were used: the first stimulus approached and the preference scores for the Red1 or Blue1 stimulus (depending on the experiment). The first stimulus approached was defined as the first choice area entered by the centroid of the chick. Preference scores were calculated as described in Exp. 1 and 2. Binomial tests were performed to analyse the number of first approaches toward each of the test stimulus. A two-way mixed-model ANOVA was used to assess the percentage of time that the chicks spent close to each stimulus, with the preference score as the dependent variable and sex (male, female) and trial (1 to 6, within subjects) as the independent variables. One-sample t-tests were used to assess whether chicks spent significantly different time near each stimulus than chance level (50%).

#### Computational methods

##### Calculating avian photoreceptor responses to colours

Avian photoreceptor responses were calculated for the feather colours of red junglefowl hens and for a library of natural spectra with the aim of relating the experimentally observed colour predispositions to the colours of the correct imprinting target (the mother hen) and the colours of the environment. Following,^78^ the photoreceptor quantum catches of an animal can be calculated using the equation:

$$QC_{i}=\int_{300}^{700}R\left(\lambda \right)S_{i}\left(\lambda \right)I\left(\lambda \right)d\lambda$$

where *QC*_*i*_ is the camera quantum catch of the sensor *i*, *R* is the spectral reflectance function of the stimulus, *S*_*i*_ is the spectral sensitivity function of the receptor *i*, and *I* is the photon flux of the illuminant, in this case set to 1 across all wavelengths. We used the receptor sensitivity functions of an average ultraviolet-sensitive avian viewer^68^^,^^71^ to calculate the quantum catches in response to our measurements of the hen feather reflectances^58^ and for the natural objects from two publicly available databases. The flower reflectance database (FReD^69^) is a large database of flower and leaf reflectance data, consisting of 2,494 spectra. 536 additional spectral reflectance functions of various types of vegetation, soil and minerals were extracted from the spectral library of the U. S. Geological Survey (USGS^70^).

The quantum catches of the four (ultraviolet, blue, green and red) colour cones *QC*_*i*_ were normalized to sum to 1, yielding relative {vs s m l} values. These {vs s m l} values were then projected to the tetrahedral colour space of birds, following the equations from^68^:

$$x=\frac{1-2s-m-u}{2}\sqrt{\frac{3}{2}}$$

$$y=\frac{-1+3m+u}{2\sqrt{2}}$$

$$z=u-\frac{1}{4}$$

where x, y, and z are Cartesian coordinates in three-dimensional (3D) space. If a colour stimulates only one class of photoreceptor, then its point (x, y, z) lies at the appropriate vertex (tip) of the tetrahedron; when all four cones are stimulated equally, in other words, the colour is achromatic, the point is at the origin (0,0,0).

$${pref}_{familiar}=\frac{{time}_{familiar}}{{time}_{familiar}+{time}_{unfamiliar}}$$

$$QC_{i}=\int_{300}^{700}R\left(\lambda \right)S_{i}\left(\lambda \right)I\left(\lambda \right)d\lambda x=\frac{1-2s-m-u}{2}\sqrt{\frac{3}{2}}y=\frac{-1+3m+u}{2\sqrt{2}}z=u-\frac{1}{4}$$

## Supplemental information

Document S1. Table S1–S15

Video S1. An example of imprinting phase followed by testing phase
