# Supplementary material for: Spontaneous biases enhance generalization in the neonate brain
Source: iScience. 2024 Jun 7;27(7):110195. doi: 10.1016/j.isci.2024.110195 (PMC11233965; doi:10.1016/j.isci.2024.110195)
Supplement: Document S1. Table S1–S15 [file mmc1.pdf]

iScience, Volume *27*

Supplemental information

Spontaneous biases enhance generalization in the neonate brain

Shuge Wang, Vera Vasas, Laura Freeland, Daniel Osorio, and Elisabetta Versace

Supplementary tables

Table S1. Related to Exp. 1A. Model comparison between linear and non-linear models for Red1-imprinting.

	AIC	Chisq	Df	p-value
Model 1 (linear)	-283.55			
Model 2 (second order polynomial)	-295.95	14.4	1	0.0001
Exponential	-280.1			
Asymptotic	-285.7			
Logistic	-284.9			
Log logistic	-283.8			

Table S2. Related to Exp. 1A. Output of the mixed-effects model examining the effect of colour distance on chicks' preference for familiarity after Red1 imprinting.

	Variance	SD		Estimate	SE	*t*-value
Subject	0.003	0.05	(Intercept)	0.58	0.011	51.58
			Poly(Colour Distance, 2)1	1.11	0.154	7.24
			Poly(Colour Distance, 2)1	-0.588	0.154	-3.83

Table S3. Related to Exp. 1B. Model comparison between linear and non-linear model for Yellow1 imprinting.

	AIC	Chisq	Df	p-value
Model 1 (linear)	-242.32			
Model 2 (second order polynomial)	-241.18	0.857	1	0.3546

Table S4. Related to Exp. 1A. Output of the mixed-effects model examining the effect of colour distance on chicks' preference for familiarity after Yellow1 imprinting.

	Variance	SD		Estimate	SE	*t*-value
Subject	0.0067	0.08172	(Intercept)	0.4865	0.022	21.69
			Colour Distance	0.0086	0.003	2.297

Table S5. Related to Exp. 2A. Model comparison between linear and non-linear models for Blue1 imprinting.

	AIC	Chisq	Df	p-value
Model 1 (linear)	-248.06			
Model 2 (second order polynomial)	-246.79	0.727	1	0.3938
Exponential	-206.9			
Asymptotic	-219.9			
Logistic	-218.8			
Log logistic	-218.3			

Table S6. Related to Exp. 2A. Output of the mixed-effects model examining the effect of colour distance on chicks' preference for familiarity after Blue1-imprinting.

	Variance	SD		Estimate	SE	*t*-value
Subject	0.0062	0.07852	(Intercept)	0.48897	0.022	22.48
			Colour Distance	0.02246	0.003	6.16

Table S7. Related to Exp. 1B. Model comparison between linear and non-linear models for Green1 imprinting.

	AIC	Chisq	Df	p-value
Model 1 (linear)	-310.40			
Model 2 (second order polynomial)	-308.41	0.0082	1	0.9277

Table S8. Related to Exp. 1B. Output of the mixed-effects model examining the effect of colour distance on chicks' preference for familiarity after Green1-imprinting.

	Variance	SD		Estimate	SE	*t*-value
Subject	0.0055	0.07421	(Intercept)	0.4897	0.0199	24.53
			Colour Distance	-0.0029	0.0033	-0.879

Table S9. Related to Exp. 2C. Model comparison between linear and non-linear model for Turquoise imprinting.

	AIC	Chisq	Df	p-value
Model 1 (linear)	-211.03			
Model 2 (second order polynomial)	-210.39	0.0082	1	0.9277

Table S10. Related to Exp. 2C. Output of the mixed-effects model examining the effect of colour distance on chicks' preference for familiarity after Turquoise imprinting.

	Variance	SD		Estimate	SE	*t*-value
Subject	0.03204	0.179	(Intercept)	0.4925	0.0094	52.20
			Colour Distance	-0.0132	0.0034	-3.837

Table S11. Related to Exp. 1C. Model comparison between linear and non-linear model for Orange imprinting.

	AIC	Chisq	Df	p-value
Model 1 (linear)	-273.24			
Model 2 (second order polynomial)	-271.62	0.3779	1	0.5387

Table S12. Related to Exp. 1C. Output of the mixed-effects model examining the effect of colour distance on chicks' preference for familiarity after Orange imprinting.

	Variance	SD		Estimate	SE	*t*-value
Subject	0.03104	0.1762	(Intercept)	0.5043	0.00839	60.05
			Colour Distance	0.0010	0.00307	0.334

Table S13. Related to colours displayed in all experiments. Colour name, RGB values and related approximate wavelength (nm) of the stimuli used in our experiments.

Colour	R	G	B	Approx. nm	Lux
Yellow1	255	255	0	580	159
Yellow2	255	230	0	588	
Yellow3	255	203	0	596	
Yellow4	255	176	0	604	
Orange	255	148	0	612	84
Red4	255	119	0	620	
Red3	255	87	0	628	
Red2	255	52	0	636	
Red1	255	0	0	645	32
Green1	26	255	0	514	129
Green 2	0	255	70	506	
Green 3	0	255	169	498	
Green 4	0	255	255	490	
Turquoise	0	222	255	482	127
Blue4	0	187	255	474	
Blue3	0	151	255	466	
Blue2	0	113	255	458	
Blue1	0	79	255	450	21

Table S14. Overview of previous spontaneous colour preference experiments in precocial birds, related to all experiments. Adapted and expanded from Ham and Osorio (2007). Extended references are provided under the table.

Species and strain	Age	Stimuli	Behaviour	Colour preferences	Reference
White Rock chicks (*Gallus gallus*)	1 day	Coloured Ostwald chips	Pecking	Preference for orange and blue over yellow and green	1
Pekin ducklings (*Anas platyrhynchos*)	1 day	Coloured Ostwald chips	Pecking	Preference for green and yellowish-green over orange and blue	1
White Rock chicks (*Gallus gallus*)	13-16 hours	Coloured spheres	Approach	Order of preference: orange, green, red, blue, yellow	2
White Rock chicks (*Gallus gallus*)	30-36 hours	Coloured spheres	Approach	Order of preference: orange, red, green, blue, yellow	2
Domestic chicks (*Gallus gallus*)	18-30 hours	Coloured balls	Approach	Equal preference for red, green, white	3
White Leghorn chicks (*Gallus gallus*)	12-24 hours	Rectangle, outline of hen	Time spent	Preference for red over white	4
White Leghorn chicks (*Gallus gallus*)	1 day	Coloured chicks	Approach	Equal preference for red, yellow, green	5
White Leghorn chicks (*Gallus gallus*)	8 days	Coloured chicks	Approach	Preference for yellow and green over red	5
Cobb chicks (*Gallus gallus*)	2 days	Coloured pen	Approach	Preference for red and yellow over blue	6
Japanese quail (*Coturnix japonica*)	4-7 days	Coloured pen	Approach	Equal preference for red and blue	6
White leghorn chicks (*Gallus gallus*)	1 day	Coloured paper disc	Approach	Preference for red over yellow or green	7
Rhode Island red chicks (*Gallus gallus*)	1 day	Flashing stimulus patterns	Approach behaviour	Initial aversion to green; equal preference for white, blue, yellow, and red	8
White leghorn chicks (*Gallus gallus*)	1 day	Coloured light	Pecking	Preference for orange-red and blue-violet	9
Bobwhite quail chicks (*Gallus gallus*)	1 day	Coloured food	Pecking	Preference in order: blue, green, yellow, red	10
ROSS 308 chicks (*Gallus gallus*)	1 day	Moving circles on computer monitors	Approach preferences (time)	Preference for red over yellow	11

1. Natural Preferences of Chicks and Ducklings for Objects of Different Colors - Eckhard H. Hess, 1956 https://journals.sagepub.com/doi/abs/10.2466/pr0.1956.2.3.477.

2. Schaefer, H.H., and Hess, E.H. (1959). Color Preferences in Imprinting Objects1. Zeitschrift für Tierpsychologie *16*, 161–172. https://doi.org/10.1111/j.1439-0310.1959.tb02051.x.

3. Smith, F.V., and Hoyes, P.A. (1961). Properties of the visual stimuli for the approach response in the domestic chick. Animal Behaviour *9*, 159–166. https://doi.org/10.1016/0003-3472(61)90004-5.

4. Smith, T.L., and Meyer, M.E. (1965). Preference of chicks in the original stimulus situation of imprinting. Psychon Sci *2*, 121–122. https://doi.org/10.3758/BF03343360.

5. Salzen, E.A., and Cornell, J.M. (1968). Self-perception and species recognition in birds. Behaviour *30*, 44–65. https://doi.org/10.1163/156853968x00171.

6. Taylor, A., Sluckin, W., and Hewitt, R. (1969). Changing colour preferences of chicks. Animal Behaviour *17*, 3–8. https://doi.org/10.1016/0003-3472(69)90105-5.

7. Salzen, E.A., Lily, R.E., and McKeown, J.R. (1971). Colour preference and imprinting in domestic chicks. Animal Behaviour *19*, 542–547. https://doi.org/10.1016/S0003-3472(71)80109-4.

8. Kovach, J.K. (1971). Effectiveness of Different Colors in the Elicitation and Development of Approach Behavior in Chicks. Behaviour *38*, 154–168.

9. Fischer, G.J., Morris, G.L., and Ruhsam, J.P. (1975). Color pecking preferences in White Leghorn chicks. J Comp Physiol Psychol *88*, 402–406. https://doi.org/10.1037/h0076227.

10. Mastrota, N.F., and Mench, J.A. (1995). Colour avoidance in northern bobwhites: effects of age, sex and previous experience. Animal Behaviour *50*, 519–526. https://doi.org/10.1006/anbe.1995.0266.

11. Freeland, L., Vasas, V., Gomes, J., and Versace, E. (2023). Assessing preferences for adult vs juvenile features in young animals: newly-hatched chicks spontaneously approach red and large stimuli. Preprint at bioRxiv, https://doi.org/10.1101/2023.02.17.528933 https://doi.org/10.1101/2023.02.17.528933.

Table S15 Related to Exp. 1, 2 and 4. For each imprinting experiment, the table reports imprinting colour, and mean, SEM and average preference during the rehearsal trails for all test colours.

Exp.	Imprinting colour									
1A	Red1	**test colours**	**R2**	**R3**	**R4**	**O**	**Y4**	**Y3**	**Y2**	**Y1**
		mean	0.445	0.524	0.541	0.601	0.633	0.642	0.640	0.617
		SEM	0.021	0.022	0.020	0.022	0.024	0.029	0.028	0.026
		rehearsal trials	0.729							
1B	Yellow1	**test colours**	**R1**	**R2**	**R3**	**R4**	**O**	**Y4**	**Y3**	**Y2**
		mean	0.568	0.501	0.534	0.575	0.520	0.524	0.491	0.485
		SEM	0.033	0.030	0.026	0.025	0.029	0.022	0.025	0.022
		rehearsal trials	0.707							
1C	Orange	**test colours**	**R1**	**R2**	**R3**	**R4**	**Y4**	**Y3**	**Y2**	**Y1**
		mean	0.486	0.513	0.503	0.497	0.541	0.484	0.504	0.507
		SEM	0.024	0.022	0.024	0.026	0.022	0.026	0.024	0.023
		rehearsal trials	0.720							
2A	Blue1	**test colours**	**B2**	**B3**	**B4**	**T**	**G4**	**G3**	**G2**	**G1**
		mean	0.509	0.521	0.543	0.620	0.605	0.616	0.648	0.659
		SEM	0.022	0.021	0.028	0.025	0.027	0.032	0.030	0.025
		rehearsal trials	0.724							
2B	Green1	**test colours**	**B1**	**B2**	**B3**	**B4**	**T**	**G4**	**G3**	**G2**
		mean	0.471	0.454	0.483	0.471	0.483	0.504	0.436	0.509
		SEM	0.022	0.028	0.029	0.021	0.026	0.021	0.023	0.020
		rehearsal trials	0.717							
2C	Turquoise	**test colours**	**B1**	**B2**	**B3**	**B4**	**G4**	**G3**	**G2**	**G1**
		mean	0.433	0.496	0.444	0.471	0.488	0.488	0.563	0.556
		SEM	0.024	0.023	0.031	0.020	0.031	0.030	0.026	0.026
		rehearsal trials	0.679							
4A	Red1	**test colours**	**R2**	**R3**	**R4**	**O**	**Y4**	**Y3**	**Y2**	**Y1**
		mean	0.435	0.576	0.558	0.712	0.711	0.628	0.699	0.636
		SEM	0.024	0.036	0.040	0.036	0.039	0.045	0.040	0.035
		rehearsal trials	0.619							
4B	Yellow1	**test colours**	**R1**	**R2**	**R3**	**R4**	**O**	**Y4**	**Y3**	**Y2**
		mean	0.642	0.623	0.615	0.683	0.686	0.641	0.678	0.491
		SEM	0.030	0.036	0.036	0.030	0.030	0.037	0.030	0.021
		rehearsal trials	0.621							
